# A Genome-Wide Association Study of Premenstrual Symptoms in Two Nordic Populations

**DOI:** 10.1016/j.bpsgos.2026.100784

**Published:** 2026-06-29

**Authors:** Elgeta Hysaj, Piotr Jaholkowski, Alexey A. Shadrin, Jacob Bergstedt, Yi Lu, Elizabeth Bertone-Johnson, Cynthia M. Bulik, Mikael Landén, Sven Sandin, Kaarina Kowalec, Sara Hägg, Arianna Di Florio, David Goldman, Peter J. Schmidt, Unnur A. Valdimarsdóttir, Ole A. Andreassen, Donghao Lu

**Affiliations:** a Institute of Environmental Medicine, Karolinska Institutet, Stockholm, Sweden; b Center for Precision Psychiatry, Division of Mental Health and Addiction, Oslo University Hospital and Institute of Clinical Medicine, University of Oslo, Oslo, Norway; c KG Jebsen Centre for Neurodevelopmental Disorders, University of Oslo, Oslo, Norway; d Department of Medical Epidemiology and Biostatistics, Karolinska Institutet, Stockholm, Sweden; e Department of Biostatistics and Epidemiology, University of Massachusetts, Amherst, Massachusetts; f Department of Health Promotion and Policy, University of Massachusetts, Amherst, Massachusetts; g Department of Psychiatry, University of North Carolina at Chapel Hill, Chapel Hill, North Carolina; h Department of Nutrition, University of North Carolina at Chapel Hill, Chapel Hill, North Carolina; i Institute of Neuroscience and Physiology, Sahlgrenska Academy, Gothenburg University, Gothenburg, Sweden; j Department of Psychiatry, Icahn School of Medicine at Mount Sinai, New York, New York; k Seaver Autism Center for Research and Treatment at Mount Sinai, New York, New York; l College of Pharmacy, Rady Faculty of Health Science, University of Manitoba, Winnepeg, Manitoba, Canada; m Department of Biochemistry and Medical Genetics, Max Rady College of Medicine, Rady Faculty of Health Sciences, University of Manitoba, Winnipeg, Manitoba, Canada; n Centre for Neuropsychiatric Genetics and Genomics, Division of Psychological Medicine and Clinical Neurosciences, Cardiff University, Cardiff, United Kingdom; o Laboratory of Neurogenetics, National Institute on Alcohol Abuse and Alcoholism, National Institutes of Health, Rockville, Maryland; p Behavioral Endocrinology Branch, National Institute of Mental Health, National Institutes of Health, Bethesda, Maryland; q Center of Public Health Sciences, Faculty of Medicine, University of Iceland, Reykjavík, Iceland; r Department of Epidemiology, Harvard T.H. Chan School of Public Health, Harvard University, Boston, Massachusetts

**Keywords:** GWAS, Heritability, MoBa, Premenstrual dysphoric disorder, Premenstrual syndrome, Women’s health

## Abstract

**Background:**

Premenstrual disorders (PMDs) are characterized by affective and physical symptoms before menses, likely due to abnormal sensitivity to normal hormone fluctuations. While sizable heritability has been indicated in twin studies, there are no genome-wide association studies (GWASs) to inform the genetic architecture of PMDs.

**Methods:**

We conducted a GWAS of 17,511 women with premenstrual symptoms (PSs) and 54,786 control women of European ancestry from 2 Nordic population-based cohorts. PSs were assessed using questionnaires or identified as a clinical diagnosis of PMD in the nationwide health care registers. A GWAS was performed in each study before meta-analysis, followed by analyses of single nucleotide polymorphism (SNP)–based heritability (*h*^*2*^_SNP_) and genetic correlations to psychosocial and gynecological phenotypes.

**Results:**

In the meta-analysis, 1 locus at 12p13.3 (rs758170, *CACNA1C*, *p* = 1.53 × 10^−8^, odds ratio [OR] = 0.93, 95% CI [0.90 to 0.95]) was associated with PSs; while the effect sizes were comparable between cohorts (LifeGene OR = 0.95 vs. MoBa [The Norwegian Mother, Father and Child Cohort Study] OR = 0.92, *p* for heterogeneity = .59), the association was not significant in LifeGene (*p* = .242). Moreover, we identified 6 loci with borderline significance, 3 of which were nominally significant in both cohorts (rs76665457, rs147346386, and rs4773561). The SNP-based heritability was estimated as 0.072 (SE = 0.01, *p* = 2.46 × 10^−12^). The strongest correlation was with major depression (*r*_g_ = 0.62, 95% CI = [0.49 to 0.74], *p* = 3.04 × 10^−22^).

**Conclusions:**

This study provides initial genetic insights into the biology of PSs by identifying an SNP associated with PSs and genetic correlations with a range of psychosocial and gynecological traits. If confirmed in larger independent populations, these findings may advance our understanding of the underlying mechanisms of PMDs.

## Plain Language Summary

We studied the genetics of premenstrual symptoms (PSs) in 72,297 European women from 2 large Nordic cohorts, LifeGene and MoBa, and compared women with PSs with control women. Our goal was to better understand the genetic architecture of PSs and their relationship to psychiatric conditions. We identified 1 genome-wide significant genetic locus at 12p13.3 (rs758170, *p* = 1.53 × 10^−8^, OR = 0.93, 95% CI [0.90 to 0.95]). We also found positive genetic correlations between PSs and all considered psychiatric disorders (*r*_g_ = 0.32−0.62). These findings highlight potential underlying mechanisms and the need for larger, more diverse studies to clarify the genetic basis of premenstrual disorders.

Premenstrual disorders (PMDs), encompassing premenstrual syndrome (PMS) and the more severe premenstrual dysphoric disorder (PMDD), are characterized by substantial mood, behavioral, and physical symptoms that occur during the luteal phase of the menstrual cycle and resolve with the onset of menstruation (1). PMDs affect millions of women of reproductive age worldwide, with an estimated prevalence of 20% to 30% for PMS and 2% to 6% for PMDD (1). Both severe PMS and PMDD are accompanied by significantly impaired social activities and relationships (2, 3, 4). Although the symptoms are often limited to the days before menses, the chronic and cyclical nature of PMDs can have a significant impact on a woman’s life (5), including increasing the risk of suicidal behavior (6,7).

While epidemiological studies have highlighted potential links with other hormone-related conditions [e.g., perinatal depression (8), menopause timing and menopause symptoms (9)], experimental studies have revealed that PMDs are triggered by an abnormal, or heightened, response to normal hormone fluctuations (10,11). However, the ontogeny of the abnormal response remains unknown, impeding the development of new treatments. Thus, uncovering the underlying causes of PMDs is essential for more effective detection and intervention.

Twin studies suggest a genetic component in PMDs, with an estimated heritability of 35% to 57% for premenstrual symptoms (PSs) (12, 13, 14, 15, 16). Moreover, research into candidate genes has indicated variants in estrogen receptor genes (17), serotonin receptor 1A (18), transcription factor AP-2 beta (19), steroid-5-alpha-reductase (20), and alpha polypeptide 1 (20) in PMDD, although conflicting results have also been noted (21). A genome-wide approach permits a more comprehensive, hypothesis-free characterization of genetic factors involved in PMDs, but such an undertaking has not been performed for PMDs. Here, we conducted a genome-wide association study (GWAS) of PSs in 2 European-ancestry samples, aiming to identify the genetic architecture of PSs and relationship of PSs with other psychiatric conditions and underlying mechanisms of PMDs.

## Methods and Materials

### Study Population

We conducted a GWAS of 72, 297 European-ancestry women nested from the LifeGene cohort in Sweden and the MoBa (The Norwegian Mother, Father and Child Cohort Study) cohort in Norway.

LifeGene is a large-scale Swedish prospective cohort launched in 2009 with longitudinal follow-ups (22). It enrolled 39,862 people (24,265 women) ages 18 to 50 years who were randomly selected from the Swedish population and their household members. A thorough web-based questionnaire for collecting information on lifestyle, physical, mental, and social well-being was administered at baseline and in 5 annual follow-up cycles. Blood samples were collected during the in-person testing at baseline. Participants were linked to the national population and health registers using their unique Swedish personal identification number, a lifelong identifier assigned at birth or upon immigration to Sweden. Informed consent was obtained either electronically from all participants upon registration online or in writing at the in-person testing center. The current study was approved by the Swedish Ethical Review Authority (2021-02775).

MoBa is a population-based pregnancy cohort study conducted by the Norwegian Institute of Public Health (23, 24, 25, 26). Participants were recruited from across Norway from 1999 to 2008. Women consented to participation in 41% of pregnancies. The cohort includes approximately 114,500 children, 95,200 mothers, and 75,200 fathers. The current study is based on version 12 of the quality-assured data files released for research in January 2019. The establishment of MoBa and initial data collection were approved via a license from the Norwegian Data Protection Agency and after review of the Regional Committees for Medical and Health Research Ethics. The MoBa cohort is currently regulated by the Norwegian Health Registry Act. The current study was approved by the Regional Committees for Medical and Health Research Ethics (2016/1226/REK).

### Assessment of PSs

Both questionnaire assessments and nationwide health care registers were sourced for case assessment. Cases with PSs were defined as either having a clinical diagnosis of PMD recorded in the registers or having met the criteria for a probable PMD based on self-report questionnaires (as described in detail in LifeGene). The controls were women with no clinical diagnosis of PMDs in registers and not meeting the PMD criteria during any available questionnaire cycles.

#### LifeGene

A modified version of the Premenstrual Symptom Screening Tool (PSST) (27) was used to assess premenstrual symptoms at baseline and annual follow-ups for 5 years (28). The original PSST has been validated with a sensitivity of 79% (29). The PSST was modified to start with 3 screening questions: 1) “During most menstruation cycles during the last year, have you experienced mood changes and/or physical symptoms during the week before menstruation?” 2) “Have your premenstrual symptoms been so severe that they have affected your relationships with others or your ability to perform work or other activities?” and 3) “Are you absolutely certain that the symptoms are limited to the premenstrual period, meaning that you are always completely symptom-free approximately a week after menstruation begins?” Upon confirmation of all screening questions, participants were prompted to rate the severity of 15 physical and affective symptoms from 1 (none), 2 (moderate), 3 (considerably severe), to 4 (severe). As described elsewhere (28), participants were classified as cases if they met 1) ≥1 of 4 affective symptoms rated as considerably severe to severe and 2) ≥4 other symptoms rated as moderate to severe.

To complement the questionnaire assessment in LifeGene, for the Swedish participants, we further identified clinical diagnoses of PMDs, as described elsewhere (7). According to the Swedish guidelines, a clinical diagnosis of PMD should be based on prospective daily symptom ratings for at least 2 consecutive menstrual cycles (30). Briefly, we identified PMD diagnoses based on ICD codes (Table S1) from the National Patient Register (NPR) (1987–2023) and the Stockholm Primary Care Register (2001–2021), since 82% of the participants lived in Stockholm County. Primary care data were unavailable for residents in other counties; we also obtained information on filled prescriptions for antidepressants (Anatomical Therapeutic Chemical codes: N06AB, N06AX, N06AA) and hormonal contraceptives (G03A) with a written indication for PMD treatment from the National Prescribed Drug Register (2006–2023).

#### The Norwegian Mother, Father and Child Cohort Study

Questionnaire assessment was based on the women’s responses to the following questions at the 15th week of gestation: “Are you usually depressed or irritable before your period?” and “If yes, does this feeling disappear after you get your period?” As described elsewhere (31), the cases were defined based on the response “yes, noticeably” or “yes, very much” to the first question and “yes” to the second question. We excluded individuals whose symptoms of PMD did not resolve after the onset of menses.

Clinical diagnoses of PMDs were derived from the Primary Care Registry of Norway (2006–2023), which contains diagnoses given at the primary care level. We identified cases based on the Premenstrual Tension Syndrome (X89) diagnosis according to the International Classification of Primary Care, Second Edition (32).

### Genotyping, Quality Control, and Imputation

#### LifeGene

In LifeGene, DNA was extracted from blood samples collected at baseline. Participants from the LifeGene cohort were genotyped by 4 substudies including the current study (Table S2). Quality control (QC) was performed using the Ricopili bioinformatics pipeline for each substudy (33). Briefly, these steps included a call rate threshold of ≥0.98 for cases and controls, heterozygosity (F_HET_) within ±0.20, and exclusion of sex mismatches. Single nucleotide polymorphism (SNP) QC required a call rate of ≥0.98, missingness difference ≤0.02, and minor allele frequency (MAF) ≥0.01. Ungenotyped SNPs were then imputed based on the Haplotype Reference Consortium (HRC) reference panel (r1.1) (34) via the Sanger imputation server and pooled after removing duplicated individuals. Information on a total of 7,135,674 SNPs was available for analysis. Admixture analysis was performed using the ADMIXTURE software to estimate genetic ancestry proportions, leveraging reference populations from the 1000 Genomes Project (phase 3 v5) (35). Analysis was restricted to autosomal variants. Individuals estimated with <90% probability of European ancestry were excluded. Briefly, genotype data were available for 8826 women (36%); after excluding 862 participants who were first-degree relatives (having identity-by-descent sharing ≥0.2), had significant non-European ancestry (*n* = 896), or had no information on phenotype (*n* = 1839), 5229 participants were included in this analysis.

#### The Norwegian Mother, Father and Child Cohort Study

In MoBa, venous blood was collected from women at approximately the 15th week of gestation and immediately after giving birth. Genomic DNA was extracted and stored at the Norwegian Institute of Public Health (36). The MoBa cohort genotyping was conducted through multiple research projects over several years (37). A novel family-based pipeline (MoBaPsychGen genotype QC pipeline) was implemented to handle the relatedness structure of the MoBa dataset, while appropriately accounting for the differences resulting from array and batch effects (37). The pipeline (38) includes preimputation QC, phasing, imputation, and postimputation QC and prioritizes retaining individuals over SNPs. After QC procedure, 6,981,748 SNPs were available for further analysis. Our analysis was restricted to individuals of European ancestry, selected based on visual comparison of the first 7 genetic principal components (PCs) with PCs from 1000 Genomes phase 1 unrelated samples.

### Statistical Analysis

#### Genome-Wide Association Study

Characteristics of individuals with and without PSs (age, educational level, civil status, history of depression or anxiety disorder, age at menarche, and parity) in LifeGene and MoBa were summarized using descriptive statistics. *p* Values were calculated using χ^2^ tests.

In LifeGene, GWAS was performed using logistic regression using PLINK2 (39). Analysis was restricted to 6,508,434 genetic variants with an MAF ≥0.01 and imputation quality score (INFO) ≥0.90. In MoBa, logistic regression was used for GWAS analysis using Regenie (version 3.2.5) (40). We excluded SNPs with an imputation info score <0.80 and SNPs with minor allele count <20. For both cohorts, the estimates were adjusted for substudy membership and the top 10 PCs in model 1. To understand the potential pleiotropic effect of the top loci on other common psychiatric conditions among women with PMD, model 2 was also adjusted for history of clinically diagnosed depression and anxiety disorder (ICD codes in Table S1). Briefly, we identified depression and anxiety diagnoses recorded before PS assessment or diagnosis from the Swedish NPR in LifeGene and the Norwegian NPR in MoBa. In an additional analysis, we restricted analysis to cases confirmed by both questionnaire assessment and clinical diagnosis.

We generated the quantile-quantile (QQ) plot using the qqman (41) package in R version 4.2.2 (2022-10-31). Summary statistics from both LifeGene and MoBa were then meta-analyzed using inverse variance-weighted analysis as implemented in METAL (version 2020-05-05) (42). SNPs with *p* value < 5 × 10^−8^ were considered genome-wide significant and with *p* value < 1 × 10^−6^ were considered borderline significant. The odds ratio (OR), 95% CI, and *p* value were reported for any independent SNPs (LD *r*^2^ < 0.6) above marginal significance (i.e., lead SNPs). Functional annotation was conducted using FUMA (43) integrating expression quantitative trait loci data from brain and blood tissues.

#### SNP-Based Heritability and Genetic Correlation

Based on the summary statistics, we estimated the SNP-based heritability on the observed scale and on the liability scale (for a prevalence of 0.2) of PSs using the linkage disequilibrium score regression (LDSC) software package (version 2.0.1) (44). Several studies have suggested that PMDs are linked to a broad range of other disorders and traits (31,45,46). We used LDSC to undertake genetic correlation analyses with major psychiatric traits (e.g., major depression), gynecological conditions (e.g., endometriosis), sex hormones, and known risk factors (e.g., age at menarche) for PMDs as described in Table S3. We also examined genetic correlations with circulating hormone levels (sex hormone binding globulin, total and bioavailable testosterone), hypothesizing a null finding because PMDs are more likely related to differential sensitivity to hormonal fluctuations than to variants in hormone levels. Briefly, we used GWAS summary statistics from the UK Biobank, where the hormone levels were measured in serum samples collected at baseline from female participants (no information on menstrual phase) using standardized immunoassays, and bioavailable testosterone was calculated using the Vermeulen equation.

#### Additional Analysis

We performed complementary analyses to evaluate the consistency of genetic signals across the LifeGene and MoBa cohorts. First, we conducted cross-cohort polygenic prediction analyses by testing whether polygenic scores derived from MoBa summary statistics predicted probable cases in LifeGene across multiple association thresholds (*p* < .05, .005, and .0005). Second, we estimated the genetic correlation between the 2 cohorts to assess shared genetic architecture. Finally, consistency of effect estimates across cohorts was formally evaluated using heterogeneity statistics from the meta-analysis.

## Results

A total of 72,297 (5229 from LifeGene and 67,068 from MoBa) women were included in the final GWAS meta-analysis. Of these, 17,511 (24.2%) met the criteria for PSs (1962 [37.5%]); cases were oversampled for genotyping, while the prevalence in the whole cohort was 10.8% from LifeGene and 23.1% (*n* = 15,549) from MoBa. In LifeGene, compared with the control women, women with PSs were more likely to have lower educational attainment, not to have a partner, and to have a prior history of depression or anxiety disorder (Table 1). Similar characteristics were observed among MoBa participants.

**Table 1:** Characteristics of Women With and Without PSs in the LifeGene and MoBa Cohorts

	LifeGene			MoBa		
	Without PSs, *n* = 3267	With PSs, *n* = 1962	*p*	Without PSs, *n* = 51,519	With PSs, *n* = 15,549	*p*
Age, Years	34 ± 8.43	33 ± 7.79	–	29.47 ± 4.56	30.1 ± 4.68	–
Educational Level^a^						
Presecondary education	112 (3.42%)	48 (2.5%)	.060	1137 (2.2%)	427 (2.7%)	<.001
Secondary education	777 (23.7%)	513 (26.1%)		15,815 (30.7%)	5393 (34.7%)	
Postsecondary education	2378 (72.4%)	1401 (71.1%)		31,936 (62.0%)	8850 (56.9%)	
Unknown	12 (0.4%)	5 (0.3%)		2631 (5.1%)	879 (5.7%)	
Civil Status^a^						
Single/separated/widower	1922 (58.8%)	1228 (62.5%)	.024	915 (1.8%)	301 (1.9%)	.003
Married/cohabitant	1342 (41.0%)	733 (37.3%)		46,648 (90.5%)	13,937 (89.6%)	
Unknown	3 (0.09%)	1 (0.05%)		3956 (7.7%)	1311 (8.4%)	
Depression^b^						
No	2795 (85.6%)	1390 (70.9%)	<.001	47,111 (91.4%)	13,208 (84.9%)	<.001
Yes	472 (14.4%)	572 (29.1%)		4408 (8.6%)	2341 (15.1%)	
Anxiety Disorder^b^						
No	2738 (83.8%)	1316 (67.1%)	<.001	47,895 (93.0%)	13,882 (89.3%)	<.001
Yes	529 (16.2%)	646 (32.9%)		3624 (7%)	1667 (10.7%)	
Age at Menarche	13.39 ± 3.64	13.20 ± 3.50	–	13.05 ± 1.37	12.93 ± 1.39	–
≤10 years	82 (2.5%)	81 (4.1%)	.005	970 (1.9%)	387 (2.5%)	<.001
11–14 years	2577 (78.9%)	1552 (79.1%)		43,036 (83.5%)	13,172 (84.7%)	
≥15 years	419 (12.8%)	232 (11.9%)		6860 (13.3%)	1807 (11.6%)	
Unknown	189 (5.8%)	97 (4.9%)		653 (1.3%)	183 (1.2%)	
Parity^a^						
0	1941 (59.5%)	1196 (60.9%)	.134	29,505 (57.3%)	7611 (48.9%)	<.001
1	398 (12.1%)	247 (12.6%)		14,740 (28.6%)	4912 (31.6%)	
2	697 (21.3%)	367 (18.7%)		6031 (11.7%)	2458 (15.8%)	
≥3	231 (7.1%)	152 (7.8%)		1243 (2.4%)	568 (3.7%)	

Values are presented as mean ± SD or *n* (%). *p* Values were obtained from χ^2^ tests.

MoBa, The Norwegian Mother, Father and Child Cohort Study; PND, perinatal depression; PSs, premenstrual symptoms.

a At baseline.

b Lifetime (in LifeGene).

### GWAS Analysis

**Figure 1:**
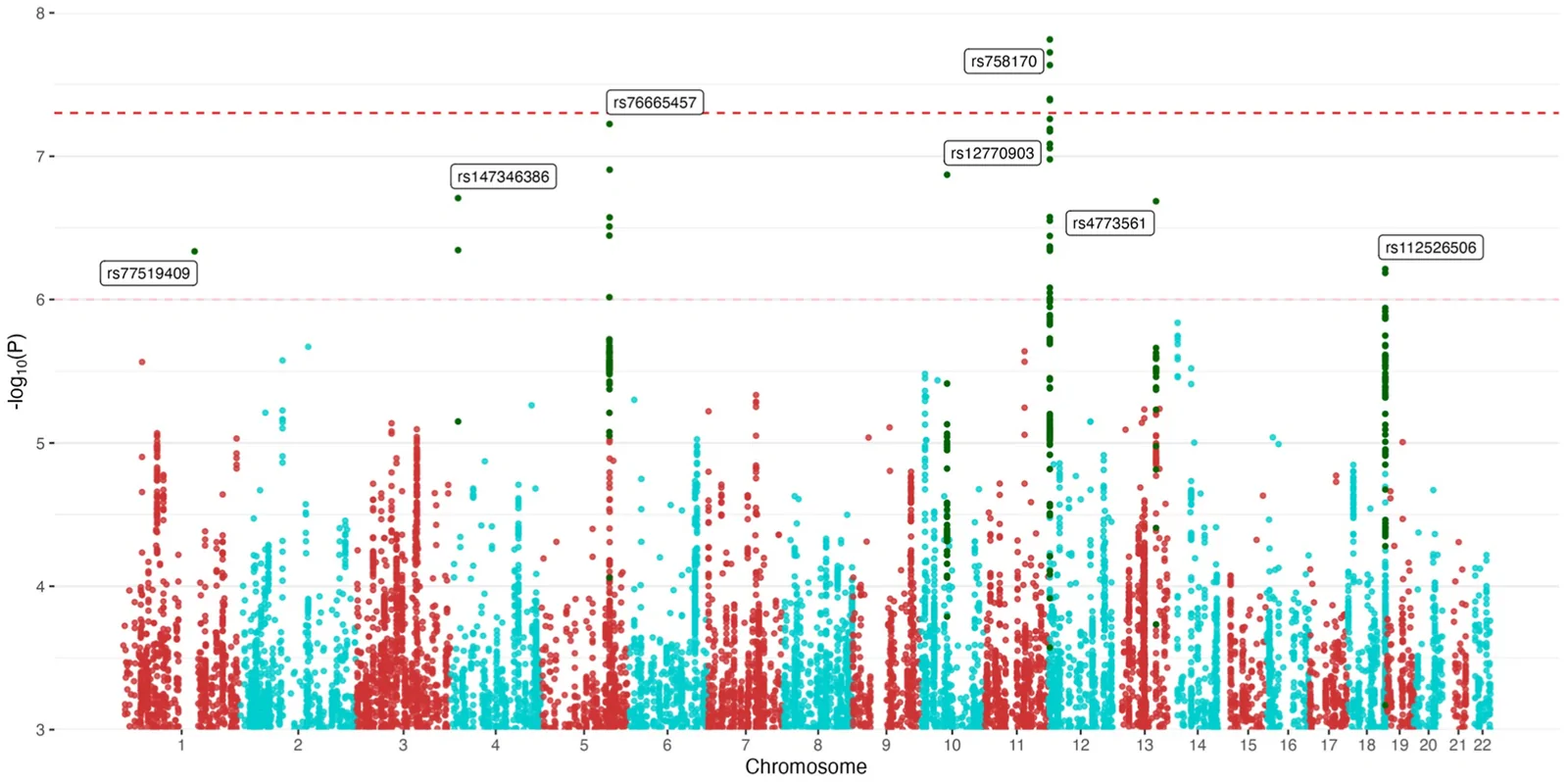
Manhattan plot from the meta-analyzed genome-wide association study of premenstrual symptoms. This analysis included 17,511 cases and 54,786 controls. The dashed red line indicates a genome-wide significant threshold of *p* = 5 × 10^−8^. The pale dashed red line indicates a suggestive significance threshold of *p* = 1.0 × 10^−6^. Green dots show SNPs in linkage disequilibrium (*r*^2^ > 0.8) with the lead SNP of each genome-wide significant or suggestive locus. The point estimates of lead SNPs are provided in Table 2. SNP, single nucleotide polymorphism.

QQ analysis of the GWAS meta-analysis indicated moderate inflation (λ = 1.075) (Figure S1). One locus at 12p13.3 was genome-wide significant (rs758170, *p* = 1.53 × 10^−8^, OR = 0.93, 95% CI [0.90 to 0.95], risk allele C) (Figure 1 and Table 2). While the effect sizes were comparable in the 2 cohorts (LifeGene OR = 0.95 vs. MoBa OR = 0.92, *p* for heterogeneity [*p*_het_] = 0.59), the association was not significant in LifeGene (*p* = .242). Moreover, 6 other loci showed a suggestive level of significance (*p* < 1.0 × 10^−6^) (Table 2), 3 of which were nominally significant in both cohorts (lead SNPs: rs76665457, rs147346386, and rs4773561).

**Table 2:** Lead SNPs Associated With Premenstrual Symptoms: A Meta-Analysis of the LifeGene and the MoBa Cohorts

Chr	Position	rsID	Gene	A1	A2	Sample	OR (95% CI)^a^	*p* Value	*p* for Heterogeneity
12	2361460	rs758170	*CACNA1C*	C	T	LifeGene	0.95 (0.86–1.04)	.242	
						MoBa	0.92 (0.90–0.95)	2.68 × 10^−8^	
						Meta-analysis	0.93 (0.90–0.95)	1.53 × 10^−8^	.5949
5	144181270	rs76665457	*CTB-85P21.1*	G	C	LifeGene	0.80 (0.63–0.99)	.032	
						MoBa	0.86 (0.81–0.91)	5.30 × 10^−7^	
						Meta-analysis	0.86 (0.80–0.86)	5.96 × 10^−8^	.5668
10	56883744	rs12770903	*PCDH15*	G	T	LifeGene	0.81 (0.62–1.02)	.066	
						MoBa	0.85 (0.80–0.91)	7.31 × 10^−7^	
						Meta-analysis	0.84 (0.78–0.90)	1.34 × 10^−7^	.7665
4	15764240	rs147346386	*CD38*	C	T	LifeGene	0.60 (0.39–0.85)	.001	
						MoBa	0.84 (0.78–0.90)	5.89 × 10^−6^	
						Meta-analysis	0.82 (0.77–0.89)	1.95 × 10^−7^	.0813
13	90887997	rs4773561	*KRT18P27*	G	A	LifeGene	0.89 (0.80–0.98)	.013	
						MoBa	0.93 (0.91–0.96)	2.96 × 10^−6^	
						Meta-analysis	0.93 (0.90–0.95)	2.06 × 10^−7^	.3235
1	151581008	rs77519409	*RP11-404E16.1*	G	A	LifeGene	–	–	
						MoBa	1.17 (1.10–1.25)	4.61 × 10^−7^	
						Meta- analysis	1.17 (1.10–1.25)	4.61 × 10^−7^	–
18	77629986	rs112526506	*KCNG2*	G	A	LifeGene	1.08 (0.95–1.23)	.183	
						MoBa	1.10 (1.06–1.15)	1.50 × 10^−6^	
						Meta- analysis	1.10 (1.06–1.14)	6.12 × 10^−7^	.8539

Lead SNPs are independent, significant SNPs that have reached either a GWAS significant *p* value < 5 × 10^−8^ or borderline significance with a *p* value < 5× 10^−6^ in the genomic risk loci. The SNPs are presented in ascending order of the *p* values. A1 indicates the risk allele, and A2 indicates the reference allele. Chr indicates position on the chromosome (GRCh37 genomic build).

GWAS, genome-wide association study; MoBa, The Norwegian Mother, Father and Child Cohort Study; OR odds ratio; SNP, single nucleotide polymorphism.

a Estimates were adjusted for 10 principal components in LifeGene and MoBa and were also adjusted for substudy membership and year of birth, respectively.

### Functional Annotation

**Figure 2:**
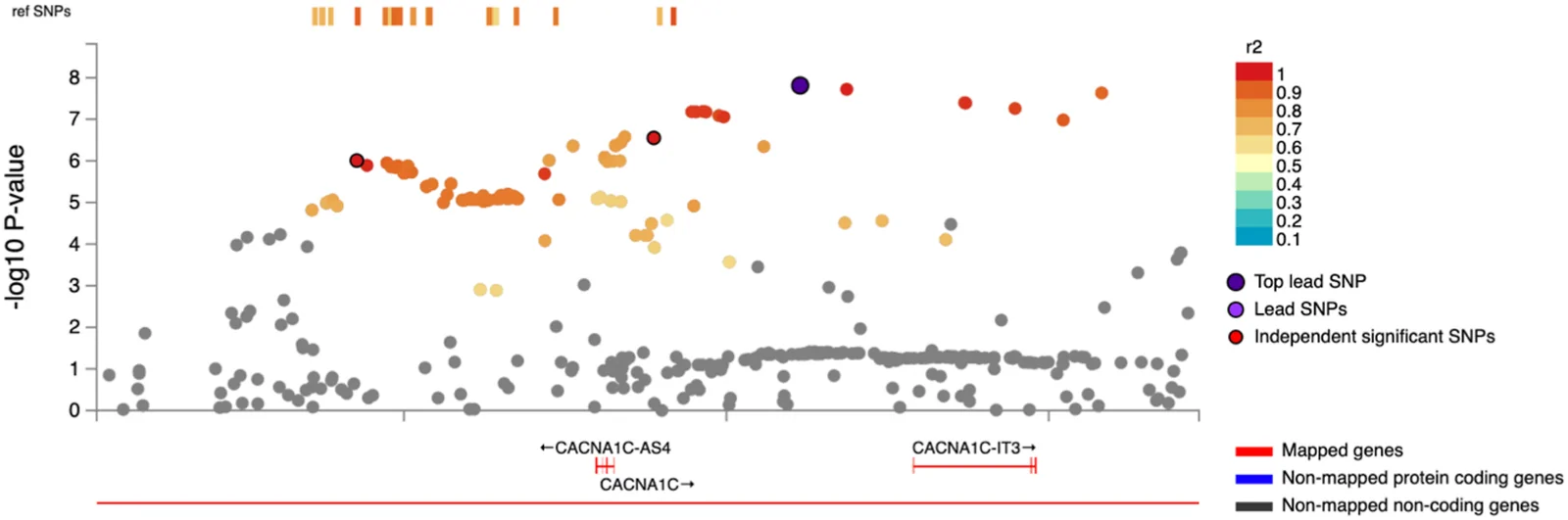
Regional plot for the lead SNP rs758170. SNPs that are not in LD of the lead SNP in the selected region are colored gray. Only SNPs that are in LD of the lead SNP are displayed in the plot. LD, linkage disequlibrium; SNP, single nucleotide polymorphism.

The top SNP, rs758170 (intron), was positionally mapped to the *CACNA1C* gene (Figure 2). FUMA indicates that the C allele associated with PS risk leads to increased expression of *CACNA1C* in the cerebellum (*p* = 2.53 × 10^−6^). Locus 10q21.1 is mapped to *RP11-478B11.2*, which is a noncoding RNA gene. The allele G of rs12770903 was associated with decreased expression of *RP11-478B11.2* in blood samples (*p* = 2.1 × 10^−4^). Other loci were not associated with differential gene expression in blood or brain tissues.

Next, we conducted several sensitivity analyses using LifeGene samples to test the robustness of our findings. To reduce the influence of comorbid psychiatric disorders, we also adjusted for depression and anxiety, yielding comparable associations for top loci (Table S5). To minimize the misclassification of probable PMD cases, we restricted analysis to cases confirmed by both questionnaire assessment and clinical diagnosis (*n* = 762). Statistically comparable point estimates were observed across cohorts, although statistical significance was not consistently observed in both datasets (Table S6).

### SNP Heritability and Genetic Correlation

**Figure 3:**
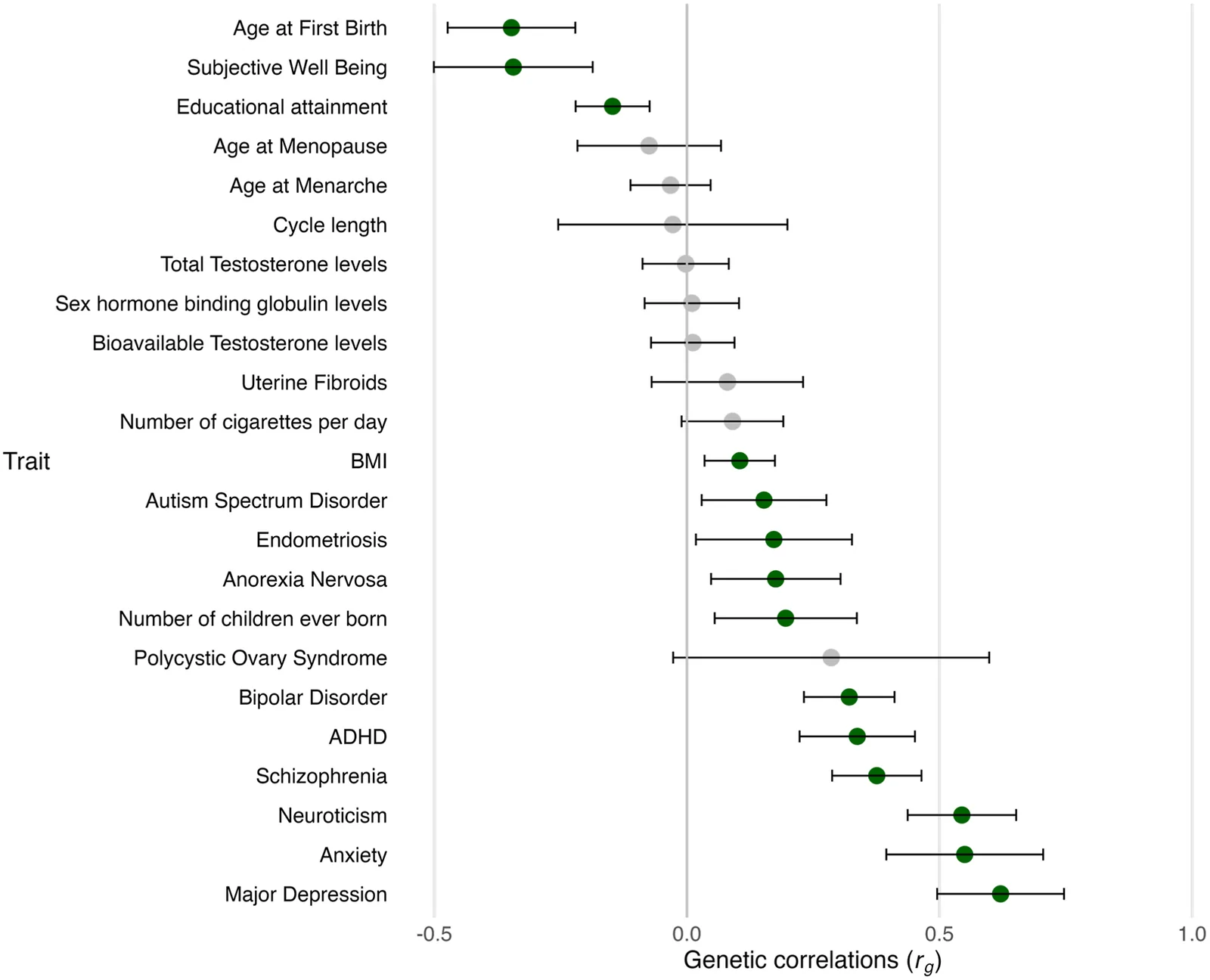
Genetic correlations (*r*_g_) between premenstrual symptoms and psychosocial characteristics, gynecological traits, and steroid hormones. Dots indicate the point estimate, while caps indicate the 95% CI. Green dots denote empirical *p* value < .05. The actual estimates are provided in Table S4. ADHD, attention-deficit/hyperactivity disorder; BMI, body mass index.

The SNP-based heritability was estimated as 0.072 (SE = 0.01, *p* = 2.46 × 10^−12^) and on the liability scale as 0.065 (SE = 0.009, *p* = 2.6 × 10^−12^). We observed significant positive genetic correlations between PSs and all considered psychiatric disorders (*r*_g_ = 0.32−0.62), with the strongest correlation being with major depression (*r*_g_ = 0.62, 95% CI [0.49 to 0.74], empirical *p* = 3.04 × 10^−22^) (Figure 3). There was a significant positive genetic correlation between PMDs and endometriosis (*r*_g_ = 0.17, 95% CI [0.01 to 0.32], empirical *p* = .029). In addition, a positive genetic correlation was found for body mass index (*r*_g_ = 0.1, 95% CI = [0.03 to 0.17], empirical *p* = .003), while significant negative genetic correlations were observed with age at first childbirth (*r*_g_ = −0.35, 95% CI = [−0.47 to −0.22], empirical *p* = 7.01 × 10^−8^), subjective well-being (*r*_g_ = −0.34, 95% CI = [−0.50 to −0.19], empirical *p* = 1.82 × 10^−5^), and educational attainment (*r*_g_ = −0.15, 95% CI = [−0.22 to −0.07], empirical *p* = 8.39 × 10^−5^) (Table S4). Finally, we did not observe any significant correlation with blood steroid hormone levels.

### Additional Analyses

To test the consistency between the LifeGene and MoBa cohorts, we performed several additional analyses. In the cross-cohort polygenic prediction analysis, we found a significantly positive association between the polygenic risk score based on the MoBa summary statistics and the probable case in the LifeGene cohort for SNPs with *p* value < .05 (OR = 1.18, *p* = 1.27 × 10^−9^). We also tested for SNPs in different *p*-value thresholds, *p* value < .005 and *p* value < .0005, and obtained similar results (OR = 1.08, *p* = .002 and OR = 1.09, *p* = .001, respectively). Moreover, the genetic correlation between the LifeGene and MoBa cohorts was estimated as 0.66 (SE = 0.38, *p* = .08). Last, the heterogeneity testing for SNPs with *p* < 1 × 10^−4^ from the MoBa cohort showed that 118 of 147 (80.3%) SNPs were statistically comparable with the corresponding point estimate from LifeGene (*p*_het_ > .05).

## Discussion

To our knowledge, this is the first GWAS focused on PSs, including 17,511 individuals with PSs and 54,786 control individuals of European genetic ancestry. We identified a genome-wide significant risk locus on 12p13.3, with comparable point estimates in LifeGene and MoBa, although the association was not statistically significant in LifeGene. Moreover, we found 6 loci with borderline significance, 3 of which were nominally significant in both cohorts. A moderate SNP-based heritability of 7.2% was observed. Genetic correlations were found between PSs and a range of psychiatric disorders, with the strongest genetic overlap noted for major depression.

The *CACNA1C* gene, implicated by linkage to rs758170, encodes the calcium voltage-gated channel subunit alpha1C, crucial for calcium channel functioning essential to neurodevelopment (47,48). While we observed genome-wide significance for this locus in the meta-analysis, the association was not significant in the better-characterized cohort, LifeGene. Because the effect sizes were highly comparable in the 2 cohorts (LifeGene OR = 0.95 vs. MoBa OR = 0.92), we speculate that the less pronounced association in LifeGene is likely due to the relatively small sample size instead of difference by phenotyping (49). Moreover, rs2370419 (chr12:2423857), an intronic variant within the *CACNA1C* gene adjacent to rs758170 (*R*^2^ = 0.10 according to LDlink), was nominally significant (*p* = .03) in LifeGene, further supporting the implication of *CACNA1C* locus. However, replication in external populations with larger sample sizes and better assessment of PMD is warranted in the future. Previous in vivo research has found that the gene *NUCB1*, encoding a calcium-binding protein, is directly involved in regulating intracellular Ca^2+^ within the endoplasmic reticulum–Golgi compartment, contributing to the abnormal response to steroid hormones in patients with PMDD (50). Moreover, studies on calcium signaling in neural cells suggest potential interactions between *KCNMA1* (calcium-dependent gene) and *NUCB1* and *CACNA1C* as part of calcium regulation pathways (51,52). Because Ca^2+^ activity in developing neural cells is modulated by various membrane receptors, including GABA (gamma-aminobutyric acid), these findings highlight promising links between calcium regulation and GABA receptor function, which has been studied widely in relation to PMDs (53,54).

*CACNA1C* has been linked to a range of psychiatric disorders (55,56). In a study investigating the effects of *CACNA1C* haploinsufficiency on mouse behavior in tests with relevance to human mood disorders, researchers found that an intronic region of *CACNA1C* was involved in mood disorder pathophysiology (57). Moreover, several association studies have linked polymorphisms in *CACNA1C* to bipolar disorder and schizophrenia (55,56), potentially through variations in mean gray matter volume and mediotemporal emotional processing (58,59). rs758170 is highly correlated (LD *r*^2^ > 0.8) with variants that have been associated with bipolar disorder (60,61), schizophrenia (62), autism, and attention-deficit/hyperactivity disorder (ADHD) (63), suggesting potentially shared disease mechanisms between PMDs and these psychiatric disorders. However, the association of rs758170 remains similar after adjustment for depression and anxiety, indicating that our finding cannot be completely explained by psychiatric comorbidities. In addition, rs758170 has been linked to lipid metabolism (64), for which several epidemiological studies have illustrated a salient relationship between adiposity and PMDs (65,66).

The *CD38* gene, linking to the borderline-significant variant rs147346386 (LifeGene OR = 0.60, *p* = .001 vs. MoBa OR = 0.84, *p* = 5.89 × 10^−6^), is involved in immune responses and has been linked to depression and anxiety (67, 68, 69). Considering the well-documented role of inflammatory processes in psychiatric disorders, the involvement of this gene is of particular significance in the context of our findings. Moreover, the *KRT18P27* gene linked to borderline-significant variant rs4773561 (LifeGene OR = 0.89, *p* = .013 vs. MoBa OR = 0.93, *p* = 2.96 × 10^−6^) has been implicated through positional gene-set enrichment to alcohol and drug dependence (70). There are no prior reports on the other borderline-significant variant, rs76665457, or the *CTB-85P21.1* gene. Future studies are needed to confirm the findings and to understand the potential mechanisms of these genetic variants and genes in relation to PMD.

While previous twin research indicates a sizable genetic contribution to PSs (16), our study represents the first attempt to estimate the genetic liability of probable PMDs. As reported in previous genetic studies of other complex traits (71,72), the SNP-based heritability estimate in our study (*h*^2^_SNP_ = 0.07) is lower than that reported in twin studies. However, this is comparable to the SNP-based heritability estimate for major depression (73). Future research should focus on larger GWASs as well as rare alleles, other alleles not well captured by GWAS, and gene-environment interplay to better capture the genetic liability of PMDs.

Extensive clinical and questionnaire-based research has consistently demonstrated a high prevalence of psychiatric comorbidities, particularly depressive and anxiety disorders, among individuals diagnosed with PMDs (1,74). In the current analyses, we found the largest and most significant genetic correlation of PMDs with major depression, consistent with the confirmed phenotypic correlation in the literature (75). Together with the observed genetic correlation with anxiety and neuroticism, it suggests that PMDs may share substantial genetic liability with internalizing disorders. That said, the abnormal sensitivity to hormone fluctuations may represent a sex-specific internalizing risk that emerges during reproductive life. It is plausible that PMDs are rather hormonally triggered manifestations of a shared underlying vulnerability with depression or internalizing disorders. In addition, other psychiatric conditions such as bipolar disorder and ADHD have been found to co-occur with PMDs (45,76). Here, we observed genetic correlations with ADHD and bipolar disorder (including bipolar I, bipolar II, and schizoaffective type), echoing the previous report on the polygenetic association with these disorders observed in MoBa (31). Future studies are needed to understand the shared phenotypic and genetic link between these disorders, particularly ADHD given the emerging data (77,78), and PMDs. Moreover, we report a significant genetic correlation between endometriosis and PSs. This is consistent with previous research reporting a phenotypic link between endometriosis and premenstrual tension (79). These data support previous research conducted on endometriosis and the phenotypic relationship with major depression and other psychiatric disorders, potentially explained by shared dysregulated immunologic functions (80). Finally, no genetic correlation was observed with steroid hormone levels, lending support to the notion that PMDs are thought to reflect differential sensitivity to hormonal fluctuations rather than absolute hormone levels as shown in previous experimental studies (10).

This study is strengthened by its large sample size, the inclusion of 2 Nordic cohorts with banked biosamples, and the use of rich register and questionnaire data for case identification. This study also has some limitations. First, a portion of the individuals with PSs were identified through screening tools. Although validity has varied across studies, ranging from chance level (81) to fairly good (27,82), large epidemiological cohorts (83) have shown that retrospective questionnaires can achieve meaningful positive predictive value. This enables the identification of potentially important cases while preserving the large sample sizes needed for research discovery. Similarly, prospective symptom charting, as is required to establish the diagnosis, is not feasible in GWASs, where a large sample size is needed, and may result in a high attrition rate particularly for those with severe symptoms (84). Moreover, to complement our questionnaire assessment, we used clinical diagnoses recorded from national and regional health care registers. While we lacked information on clinical diagnostic process, prospective symptom charting has been outlined in clinical guidelines in Sweden (30). Although clinical guidelines are generally well followed in the Nordic countries due to the tax-funded health care system, studies have shown that in the United States, at least few health care providers use daily symptom monitoring for the diagnosis of PMD (85). With both questionnaire assessment and register-recorded diagnoses, we still might have captured moderate/severe PMS, PMDD, and false positive cases. However, such misclassification should be nondifferential in terms of the exposure (genetic variants) and would have attenuated the associations toward the null (86). Importantly, in a sensitivity analysis, we restricted cases to those confirmed by both questionnaire assessment and clinical diagnosis, which likely reflects higher diagnostic validity. While the analysis was limited in power and the point estimates differed somewhat, the effect directions for the top SNPs were broadly consistent. Second, depressive or anxiety symptoms may bias the assessment of PSs, leading to potential misclassifications. However, comparable results for the top loci were observed after adjustment for history of depression and anxiety. Nonetheless, given the high comorbidity and shared vulnerability (75,87, 88, 89), the presence of PMD in the current GWAS of depression and anxiety disorders can be substantial, which could spuriously boost the genetic correlation observed between these conditions. That said, the similarity between the current PMD GWAS and MDD GWAS is expected, and so would be for the genetic correlations with other psychiatric traits. Strong genetic correlations with anxiety and neuroticism have been observed for both PMD and MDD (31). Third, while it is unclear whether depression/anxiety symptoms were present at the assessment of PSs, our questionnaires are not designed to capture individuals with chronic mood disorders with perimenstrual worsening, i.e., premenstrual exacerbation (PME). In the absence of an established clinical diagnosis for PME, it is also difficult to identify this diagnosis from registers. However, PME represents a group potentially sharing underlying mechanisms with PMS/PMDD, and future research is needed to understand the shared or nonshared genetic architectures between these disorders. As the current study was not designed to formally assess pleiotropy with depression and related psychiatric traits, the specificity of the identified associations to PSs remains uncertain. Future studies incorporating formal cross-trait analyses will be needed to distinguish shared from trait-specific genetic effects. Fourth, due to the small numbers of participants, we removed participants of non-European ancestry. Future studies should include diverse ancestral backgrounds to aim for generalizable results across different groups, thereby making the findings relevant and potentially beneficial to a wider population, as the lack of replication in the current study is a limitation. Moreover, the difference in phenotyping impacts the prevalence rate between cohorts, enclosing a wider phenotypic heterogeneity. Finally, the phenotyping difference (different questionnaires to assess PSs) between the 2 cohorts and the relatively small sample size in LifeGene make it difficult to replicate the MoBa findings with the same level of statistical significance. However, the additional analyses of cross-cohort polygenic prediction, positive but nonsignificant genetic correlation between LifeGene and MoBa, as well as 80% to top SNPs in MoBa showing statistical comparable results in LifeGene lend further support to the cross-cohort phenotype compatibility and common genetic signals. Given differences in phenotype definition across cohorts, including both self-reported symptom measures and diagnostic phenotypes, our findings should be interpreted cautiously. The identified signals may reflect genetic influences on PSs captured in population-based samples and may also partially overlap with broader affective or internalizing liability. That said, our study underscores the need for larger studies with improved and harmonized phenotyping in the future.

### Conclusions

In this first genome-wide association meta-analysis of PSs based on retrospective questionnaire assessment and register-based diagnoses, we report a significant locus and genetic correlations with a range of psychosocial and gynecological phenotypes. The identified genetic markers may help advance our understanding of the underlying mechanisms, which may further inform the development of early detection and clinical management for PMDs. Future studies with larger, more diverse samples and cases confirmed with prospective symptom charting are needed to further understand the genetic influence on PMDs and potentially the mechanisms of mood-regulating effects of sex hormones.

## Supplementary Material

Figure S1 and Tables S1–S6

Key Resources Table
