# Supplementary material for: A Genome-Wide Association Study of Premenstrual Symptoms in Two Nordic Populations
Source: Biol Psychiatry Glob Open Sci. 2026 Jun 29;6(5):100784. doi: 10.1016/j.bpsgos.2026.100784 (PMC13499404; doi:10.1016/j.bpsgos.2026.100784)
Supplement: Supplementary file 1 [file mmc1.pdf]

SUPPLEMENTARY INFORMATION

Genome-Wide Association Study of Premenstrual Symptoms in Two Nordic Populations

Hysaj *et al.*

Figure S1. QQ-plot of GWAS meta-analysis summary statistics.

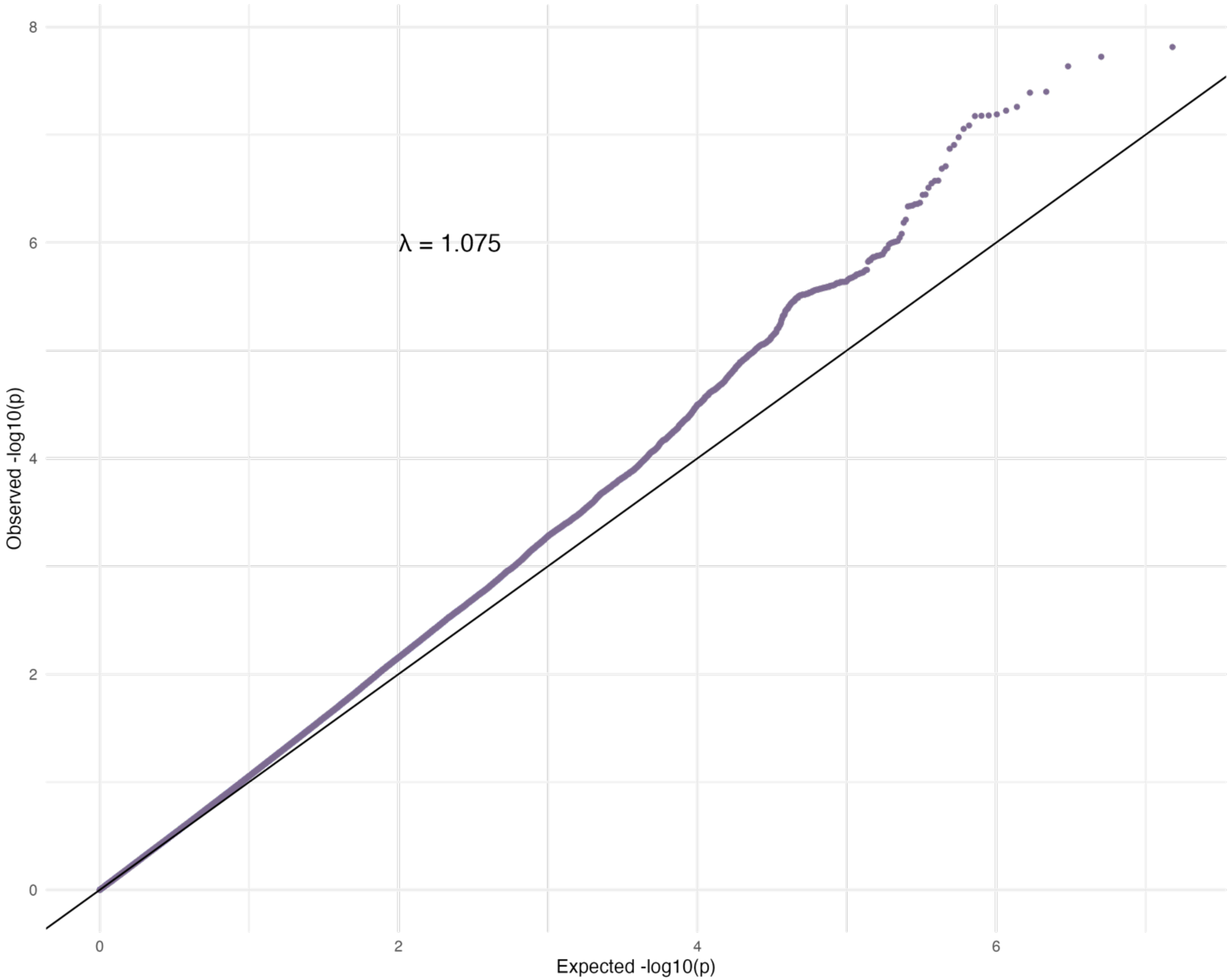

Table S1. Codes used for identifying premenstrual disorders, depression, and anxiety from Swedish registers.

Diagnosis	Source	Type	Codes
Premenstrual disorders	NPR	ICD-10	N943 ,625E
	MDR	ATC	N06AB, N06AX, N06AA, G03A<br><br>Associated with a Swedish written indication for PMDs:<br><br>”PMS”,<br>"PREMENSTRUELLT SYNDROM",<br>"PREMENSTRUELLTDYSFORISKT SYNDROM",<br>"PREMENSTRUELL DYSFORI", "PMD", "PMDD", "PMDS",<br>"MENS"
Depression	NPR	ICD-9	296
		ICD-10	F32-F33
Anxiety	NPR	ICD-9	300
		ICD-10	F41

Abbreviations: ICD, International Classification of Diseases; NPR, National Patient Register; MDR, National Prescribed Drug Register; PMDs, Premenstrual Disorders.

Table S2. Genotyping information for the sub-studies in LifeGene.

Sub-study	Genotyping facility	Chip
**HER** (Hormones, Emotions, Reproduction)	SNP&SEQ Technology Platform (Uppsala, Sweden)	Illumina Infinium GSA- MD version 3
**S3** (Swedish Schizophrenia Study)	Broad Institute (Cambridge, MA, USA)	Affymetrix 5.0, Illumina Omni Express
**ANGI** (Anorexia Nervosa Genetics Initiative)	Broad Institute (Cambridge, MA, USA)	GSAMD-24v1
**PAGES** (Population-Based Autism Genetics and Environmental Study)	Icahn School of Medicine at Mount Sinai (New York, NY, USA)	Illumina HumanOmniExpressExome BeadChip

Table S3. GWAS summary statistic sources of psychiatric traits, gynecologic conditions, sex hormones, and known risk factors for premenstrual disorders used for the analysis of genetic correlation.

Trait	Reference
Schizophrenia	PMID: 35396580
Neuroticism	PMID: 29942085
Autism spectrum disorder	PMID: 30804558
Attention deficit hyperactivity disorder (ADHD)	PMID: 36702997
Anxiety	PMID: 26754954
Anorexia nervosa	PMID: 31308545
Major depression	PMID: 29700475
Bipolar disorder	PMID: 31043756
Educational attainment	PMID: 35361970
Subjective well being	PMID: 27089181
Number of cigarettes per day	PMID: 31036639
BMI	PMID: 30124842
Age at menarche	PMID: 29892013
Menstrual cycle length	PMID: 30202859
Age at first birth	PMID: 26787164
Number of children ever born	PMID: 26751176
Age at menopause	PMID: 37543033
Polycystic ovary syndrome (PCOS)	PMID: 34791234
Endometriosis	PMID: 36914876
Uterine fibroids	PMID: 31649266
Total testosterone levels	PMID: 32042192
Sex hormone binding globulin levels	PMID: 32042192
Bioavailable testosterone levels	PMID: 32042192

Table S4. Genetic correlations between disordered premenstrual symptoms and psychosocial, gynecological traits, and steroid hormones. The traits are presented in descending order of the genetic correlation coefficient.

**Trait**	**rg**	**Lower CI**	**Upper CI**	**SE**	**p-value**
Age at first birth	-0.35	-0.47	-0.22	0.06	7.0199e-08
Subjective well being	-0.34	-0.50	-0.19	0.08	1.8274e-05
Educational attainment	-0.15	-0.22	-0.07	0.04	8.3924e-05
Age at menopause	-0.07	-0.22	0.07	0.07	0.3029
Age at menarche	-0.03	-0.11	0.05	0.04	0.4219
Cycle length	-0.03	-0.25	0.20	0.12	0.8094
Total testosterone levels	0.00	-0.09	0.08	0.04	0.9541
Sex hormone binding globulin levels	0.01	-0.08	0.10	0.05	0.8391
Bioavailable testosterone levels	0.01	-0.07	0.09	0.04	0.7827
Uterine fibroids	0.08	-0.07	0.23	0.08	0.2946
Number of cigarettes per day	0.09	-0.01	0.19	0.05	0.079
BMI	0.10	0.03	0.17	0.04	0.0033
Autism spectrum disorder	0.15	0.03	0.28	0.06	0.0154
Endometriosis	0.17	0.02	0.33	0.08	0.0289
Anorexia nervosa	0.18	0.05	0.30	0.07	0.0071
Number of children ever born	0.20	0.05	0.34	0.07	0.0064
Polycystic ovary syndrome	0.29	-0.03	0.60	0.16	0.0736
Bipolar disorder	0.32	0.23	0.41	0.05	1.9643e-12
ADHD	0.34	0.22	0.45	0.06	6.8362e-09
Schizophrenia	0.38	0.29	0.46	0.05	8.4847e-17
Neuroticism	0.54	0.44	0.65	0.05	2.7732e-23
Anxiety	0.55	0.39	0.71	0.08	3.9104e-12
Major depression	0.62	0.50	0.75	0.06	3.0436e-22

rg, genetic correlation; *lower_ci*, lower confidence interval; *upper_ci*, upper confidence interval; *se*, standard error. This table is also presented in Figure 2.

Table S5. Lead SNPs associated with distressing premenstrual symptoms in the meta-analysis: additionally adjusted for depression and anxiety.

Chr	Position	rsID	Gene	A1	A2	Sample	OR (95% CI) [a]	*P-value*
12	2361460	rs758170	*CACNA1C*	C	T	LifeGene	0.93 (0.86–1.02)	0.169
						MoBa	0.92 (0.90–0.95)	9.65×10^{-8}
						Meta-analysis	0.92 (0.90–0.95)	3.77×10^{-8}
5	144181270	rs76665457	*CTB-85P21.1*	G	C	LifeGene	0.81 (0.67–0.97)	0.026
						MoBa	0.86 (0.81–0.91)	3.56×10^{-7}
						Meta-analysis	0.85 (0.81–0.90)	3.44×10^{-8}
10	56883744	rs12770903	*PCDH15*	G	T	LifeGene	0.81 (0.66–1.00)	0.051
						MoBa	0.85 (0.80–0.91)	7.58×10^{-7}
						Meta-analysis	0.85 (0.80–0.90)	1.16×10^{-7}
4	15764240	rs147346386	*CD38*	C	T	LifeGene	0.65 (0.50–0.84)	0.001
						MoBa	0.84 (0.78–0.91)	1.34×10^{-5}
						Meta-analysis	0.83 (0.77–0.89)	4.81×10^{-7}
13	90887997	rs4773561	*KRT18P27*	G	A	LifeGene	0.88 (0.81–0.97)	0.011

Chr	Position	rsID	Gene	A1	A2	Sample	OR (95% CI) [a]	*P-value*
						MoBa	0.93 (0.91–0.96)	2.57×10^{-6}
						Meta-analysis	0.93 (0.90–0.95)	1.63×10^{-7}
1	151581008	rs77519409	*RP11-404E16.1*	G	A	LifeGene	-	-
						MoBa	1.18 (1.10–1.26)	2.33×10^{-7}
						Meta- analysis	1.18 (1.10–1.26)	2.33×10^{-7}
18	77629986	rs112526506	*KCNG2*	G	A	LifeGene	1.08 (0.95–1.23)	0.205
						MoBa	1.10 (1.06–1.15)	6.44×10^{-7}
						Meta- analysis	1.10 (1.06–1.15)	2.92×10^{-7}

Chr chromosome, *SNP* single-nucleotide polymorphism, *A1* Risk allele, *A2* reference allele*, OR odds ratio, CI* confidence interval.
[a]Estimates were adjusted for 10 principal components, depression and anxiety disorder in LifeGene and MoBa; and were additionally adjusted for the sub-study membership and year of birth, respectively.

Table S6. Lead SNPs associated with disordered premenstrual symptoms in the LifeGene cohort: restricting cases to confirmed by both questionnaire assessment and clinical diagnosis.

Chr	Position	rsID	Gene	A1	A2	Sample	OR (95% CI) [a]	*P-value*
12	2361460	rs758170	*CACNA1C*	C	T	LifeGene	1.03 (0.88–1.21)	0.635
						MoBa	0.80 (0.68–0.94)	0.007
						Meta-analysis	0.91 (0.82–1.02)	0.130
5	144181270	rs76665457	*CTB-85P21.1*	G	C	LifeGene	0.91 (0.65–1.26)	0.575
						MoBa	1.16 (0.82–1.66)	0.387
						Meta-analysis	1.02 (0.80–1.30)	0.854
10	56883744	rs12770903	*PCDH15*	G	T	LifeGene	0.98 (0.68–1.41)	0.917
						MoBa	0.65 (0.47–0.91)	0.013
						Meta-analysis	0.78 (0.61–1.00)	0.057
4	15764240	rs147346386	*CD38*	C	T	LifeGene	0.71 (0.45–1.11)	0.227
						MoBa	0.96 (0.59–1.54)	0.876
						Meta-analysis	0.81 (0.59–1.13)	0.231
13	90887997	rs4773561	*KRT18P27*	G	A	LifeGene	0.82 (0.70–0.96)	0.016
						MoBa	0.82 (0.70–0.97)	0.021

Chr	Position	rsID	Gene	A1	A2	Sample	OR (95% CI) [a]	*P-value*
						Meta-analysis	0.82 (0.73–0.92)	9.05 x 10^{-3}
1	151581008	rs77519409	*RP11-404E16.1*	G	A	LifeGene	-	-
						MoBa	1.16 (0.80–1.68)	0.415
						Meta- analysis	1.16 (0.80–1.68)	0.415
18	77629986	rs112526506	*KCNG2*	G	A	LifeGene	1.06 (0.85–1.33)	0.575
						MoBa	1.02 (0.80–1.30)	0.839
						Meta- analysis	1.04 (0.88–1.23)	0.584

Chr chromosome, *SNP* single-nucleotide polymorphism, *A1* Risk allele, *A2* reference allele*, OR odds ratio, CI* confidence interval.
In total, 762 cases and 54,780 controls were included in the meta-analysis.
[a]Estimates were adjusted for 10 principal components in LifeGene and MoBa; and were additionally adjusted for the sub-study membership and year of birth, respectively.
